# Indole Alkaloids from Plants as Potential Leads for Antidepressant Drugs: A Mini Review

**DOI:** 10.3389/fphar.2017.00096

**Published:** 2017-02-28

**Authors:** Hazrulrizawati A. Hamid, Aizi N. M. Ramli, Mashitah M. Yusoff

**Affiliations:** Faculty of Industrial Sciences & Technology, Universiti Malaysia PahangGambang, Malaysia

**Keywords:** indole alkaloids, antidepressant, *Passiflora incarnata* L, *Mitragyna speciosa*, *Piper methysticum* G. Forst, *Valeriana officinalis* L

## Abstract

Depression is the most common illness observed in the elderly, adults, and children. Antidepressants prescribed are usually synthetic drugs and these can sometimes cause a wide range of unpleasant side effects. Current research is focussed on natural products from plants as they are a rich source of potent new drug leads. Besides *Hypericum perforatum* (St. John’s wort), the plants studied include *Passiflora incarnata* L. (passion flower), *Mitragyna speciosa* (kratom), *Piper methysticum* G. Forst (kava) and *Valeriana officinalis* L. Harman, harmol, harmine, harmalol and harmaline are indole alkaloids isolated from *P. incarnata*, while mitragynine is isolated from *M. speciosa*. The structure of isolated compounds from *P. methysticum* G. Forst and *V. officinalis* L. contains an indole moiety. The indole moiety is related to the neurotransmitter serotonin which is widely implicated for brain function and cognition as the endogenous receptor agonist. An imbalance in serotonin levels may influence mood in a way that leads to depression. The moiety is present in a number of antidepressants already on the market. Hence, the objective of this review is to discuss bioactive compounds containing the indole moiety from plants that can serve as potent antidepressants.

## Introduction

According to the World Health Organization, depression affects an estimated 350 million people worldwide (Organization, 2017). Patients with depression indicate symptoms of anxiety disorders and accompanied with an inability to experience pleasure and interest, loss of concentration, self-doubt, social anxiety, sleep and appetite disorder (Namola et al., 2015). The main factors that cause depression are chemicals or hormones imbalance in the brain. The main hormone associated with depression is serotonin. Other hormones are norepinephrine and dopamine (Yi et al., 2008). These hormones are necessary for normal brain function and to control feelings. The destruction of these hormones may cause chemical imbalance in the brain resulting in depression.

Depression can be treated depending on its severity, by psychotherapy or medication. Antidepressants are the main types of medication used to treat depression. There are many different types of antidepressant drugs available, and they differ only in the way they act on the brain, their cost, and their side effects profile. In the first line treatment, most patients are either prescribed a tricyclic antidepressant (TCA) or a selective serotonin reuptake Inhibitor (SSRI; McCarthy et al., 2016). The drugs that are commonly used for anxiety treatments are benzodiazepines. Although there are many antidepressant drugs in the market used to treat depression, the after effects of using these drugs are of great concern (Binfaré et al., 2009). An alternative therapy of depression is the use of herbal medicines (Fajemiroye et al., 2016). The use of herbal extracts is gaining wider acceptance among the medical profession and by patients. The majority of herbal remedies utilized for the treatment of depression are crude or semipurified extracts (Calixto et al., 2000; Carlini, 2003; Guan and Liu, 2016).

There is scarcity in reports on research involving the active principle capable of inducing activity on the central nervous system (CNS). A review by Carlini (2003) includes information of only on psychoanaleptic, psycholeptic, and psychodysleptic effects. A recent review by Guan and Liu (2016) discussed the structure–activity relationship of the antidepressant effects of flavonoids isolated from natural and synthetic sources. Synthetic indole alkaloids, their activity, and potential use in medicine have already been reviewed in several articles (de Sa et al., 2009). However, no review paper has been published correlating plant indole alkaloids isolated with antidepressant activity. This review provides information on the potential of natural indole alkaloids for the treatment of neurological disorder, structure-activity relationship studies, and extent these to other bioactive metabolites as potential antidepressant drug leads from the perspective of chemical structure. It is compiled through bibliographic investigation of scientific journals and relevant literature identified through Web of Science electronic databases.

## Antidepressant Plants

This review article deals with plants possessing activity on the CNS. Although many types of plants fall into this category, we will highlight only plants which exhibit antidepressant properties. Two plants that contain indole alkaloids are *Passiflora incarnata* L. (passion flower) and *Mitragyna speciosa* (Korth.) Havil (kratom), while the other two plants that did not show the presence of indole alkaloids are *Piper methysticum* G. Forst (kava) and *Valeriana officinalis* L., deserve special attention. Chemical structure of isolated compounds from these plants can be used as the basis for the development of new drugs.

*Passiflora incarnata* and other species such as *P. alata* Curtis, *P. coerulea* L. and *P. edulis* Sims are widely used as sedative in traditional medicine in most European countries and in America (Houghton and Seth, 2003). The structure of benzodiazepines drugs consists of a benzene ring fused to a diazepine system comprising a seven-membered heterocyclic moiety with two nitrogen atoms in positions 1 and 2 of the ring. Indole alkaloids isolated from *P. incarnata* namely harman, harmol, harmine, harmalol and harmaline consist of a benzene ring fused to a five membered heterocycle containing one nitrogen atom.

Several studies have indicated that *P. incarnata* has a pharmacological profile similar to benzodiazepines and acts through gamma-aminobutyric acid (GABA) receptors (Jawna-Zboiñska et al., 2016). The leaves of *M. speciosa* have been used as a traditional medicine to treat diarrhea, diabetes and to improve blood circulation (Vicknasingam et al., 2010). Mitragynine is the major indole alkaloid present in *M. speciosa* with its analogs, speciogynine, paynantheine and speciociliatine (León et al., 2009). Two studies conducted on aqueous extract and alkaloidal extract of *M. speciosa* induced antidepressant-like effect on mouse models of behavioral despair (Kumarnsit et al., 2007). A study conducted by Idayu et al. (2011) on mitragynine shows the effect of antidepressants in animal behavioral model of depression through the interaction with the hypothalamic-pituitary-adrenal (HPA) axis in the neuroendocrine system (Idayu et al., 2011).

*Piper methysticum* G. Forst is consumed as a drink called kava which induces a pleasant mental state toward feeling cheerful while reducing fatigue and anxiety (Bilia et al., 2002). The study shows that most of the pharmacological activities are retained from the lipid-soluble extract compared to aqueous extract. The lipid-soluble extract contains at least seven pyrones, known as kavalactone. Kavalactones generally interacts with the dopaminergic, serotonin, gamma aminobutyric acid (GABA), glutamatergic neurotransmissions, prevents monoamine oxidase B (MOB) and also provide a variety of effects on ion channels (Grunze et al., 2001). Dihydromethysticin is one of the six major kavalactones found in the kava plant. The structure of dihydromethysticin comprises an arylethylene-α-pyrone bound to an indole-like moiety comprising two oxygens instead of nitrogens. It contributes toward anxiolytic activity and act as antidepressant drugs. Double-blind placebo-controlled studies demonstrated that kavalactones effect anxiolytic activities without depressing mental and motor functions and improves the quality of sleep. Kavalactones is an alternative to replace the usage of benzodiazepines in depression therapy (Malsch and Kieser, 2001).

*Valeriana officinalis* L., also known as valerian is widely used by many countries as a sedative, anticonvulsant, for hypnotic effects, and anxiolytic activity (Ghaderi and Jafari, 2014). Valerenic acid and valepotriates have been reported as active ingredients in pharmaceutical preparations and valerian commercial crude extracts have recorded use in many countries (Bos et al., 2002). Valepotriates which comprise triesters of polyhydroxycyclopenta-(c)-pyrans with the carboxylic acids: acetic, valeric, isovaleric, α-isovaleroxy-isovaleric, β-methylvaleric, β-acetoxy-isovaleric, β-hydroxyisovaleric and β-acetoxy-β-methylvaleric acid, are used as sedatives. Valepotriates are unstable, thermolabile and decompose rapidly under acidic or alkaline conditions in water, as well as in alcoholic solutions (Bos et al., 2002). Valepotriates are useful in improving animal and human conditions during benzodiazepine withdrawals (Poyares et al., 2002).

Although there are a variety of different chemical constituents, the mechanism of action is reported as interaction of valerian with the GABA system in the brain through inhibition of GABA transaminase, the interaction with the GABA receptor/benzodiazepine and interference in uptake and intake recruitment of GABA in synaptosomes (Sichardt et al., 2007). Various models have been utilized to investigate antidepressant effects of plants. Pharmacodynamic models include *in vitro*, *in vivo* and clinical models were utilized to assess the effects (**Table 1**). A majority of the selected plants species reviewed were studied at *in vivo* level, a few study *in vitro* and only kava had undergone clinical trials. In all of the cases, more research needs to be carried out to establish the active compounds, most effective dose and to determine whether this varies between different types of depressions.

**Table 1:** Information of pharmacology studies of reviewed plants.

Family and scientific name	Part used	Pretreatment of material	Extraction method	Active compound/Fraction/extract	Model	Duration of administration	Dosage concentration	Reference
*Passiflora incarnate In vitro*	Aerial	Dry	Extraction in 60% ethanol	Ethanol extract	Serotonin re-uptake in rat	30 min	50 μg/ml	Fiebich et al., 2011
*In vivo*	Aerial	Dry	Extraction in 60% ethanol	Ethanol extract	Forced swimming test Open Field test	24 h	45 mg/kg	Fiebich et al., 2011
*In vitro*	NS	NS	Ethanol 60%	Ethanol extract	Spatial memory in water maze, Levels of amino acids, monoamines, in select brain regions	7 weeks	30, 100, or 300 mg/kg body weight/day	Jawna-Zboiñska et al., 2016
*Mitragyna speciosa* (Korth.) Havil *In vivo*	Leaves	Dry at 45–50°C	Macerated with absolute methanol for 72 h	Mitragynine	Forced swim test Tail suspension test	7 days	10 mg/kg and 30 mg/kg	Idayu et al., 2011
*In vivo*	Leaves	Dry for 2 weeks	Macerated with methanolic for 20 h	Methanolic extract	Apomorphine-induced climbing behavior, Haloperidol-induced catalepsy, Ketamine-induced social withdrawal tests	60 min	50–500 mg/kg	Vijeepallam et al., 2016
*Piper methysticum* G. Forst *Clinical trial*	NS	NS	NS	Extract	Randomized, Placebo-controlled, Double-blind study	5 weeks	50–300 mg/day	Malsch and Kieser, 2001
*Clinical trial*	Rhizome	NS	NS	Aqueous extract	The Kava Anxiety Depression Spectrum Study	4 weeks	1.8 g tablet, three times/day	Sarris et al., 2009
*Valeriana officinalis* L *In vivo*	Root	Dry	Percolation	Extrat 45% methanol, 70 and 30 % ethanol Extract phytofin Valerian 368	Forced swimming Horizontal wire test	16 days	100–500 mg/kg	Hattesohl et al., 2008


*NS, not stated.*

## Indole Alkaloids

Indole alkaloids have a bicyclic structure, consisting of a six-membered benzene ring fused to a five-membered nitrogen-containing pyrrole ring. This pyrrole ring with nitrogen atom gives rise to the basic properties of indole alkaloids that make them particularly pharmacologically active (El-Sayed and Verpoorte, 2007). Indole alkaloids are widely distributed in plants belonging to the families Apocynaceae, Loganiaceae, Rubiaceae, and Nyssaceae. Important indole alkaloids which have been isolated from plants include the antihypertensive drug, reserpine from *Rauvolfia serpentina* (Sagi et al., 2016) and the powerful antitumor drugs, vinblastine and vincristine from *Catharanthus roseus* (El-Sayed and Verpoorte, 2007). Studies on the effectiveness of indole alkaloids in treating depression is not new and has been conducted since 1952, but currently very little attention has been given by the scientific community to the benefits of the therapeutic usefulness of plants endowed with antidepressant properties.

Indole alkaloids are often associated with the function of G-protein receptors, in particular for neuronal signal transmission through receptors for serotonin (5-HT/hydroxytryptamine). Apart from the hydrogen donor via free N-H, the presence of π-electrons density contributes to the highest occupied molecular orbital (HOMO) energy of the planar indole skeleton. This allows interaction with nucleobases, in particular protonated atom as well as target proteins (de Sa et al., 2009). The chemical structure of the neurotransmitter serotonin is based on electron-rich aromatic indole ring. The presence of nitrogen atom in indole ring is to maintain the aromatic system and makes binding N-H acidic rather than nitrogen basic. The indole ring is able to form hydrogen bonds through the N-H moiety and π–π stacking or cation–π interactions, via the aromatic moiety (Shimazaki et al., 2009). Hydrophobicity of indole rings is almost the same as the phenyl subunit and less hydrophobic than the classic isosteric benzothiophene and benzofuran ring. The N-H indole group play a decisive role in the interaction with target bioreceptor while synthesized benzothiophene and benzofuran derivatives show moderate to limited affinity for the target bioreceptor (de Sa et al., 2009). Reserpine is one of the examples of indole alkaloids isolated during the last 60 years which show sedative action on the CNS. Incidentally, two chemicals, viz., tryptamine and serotonin found in the brain are also indole alkaloid derivatives.

**FIGURE 1:**
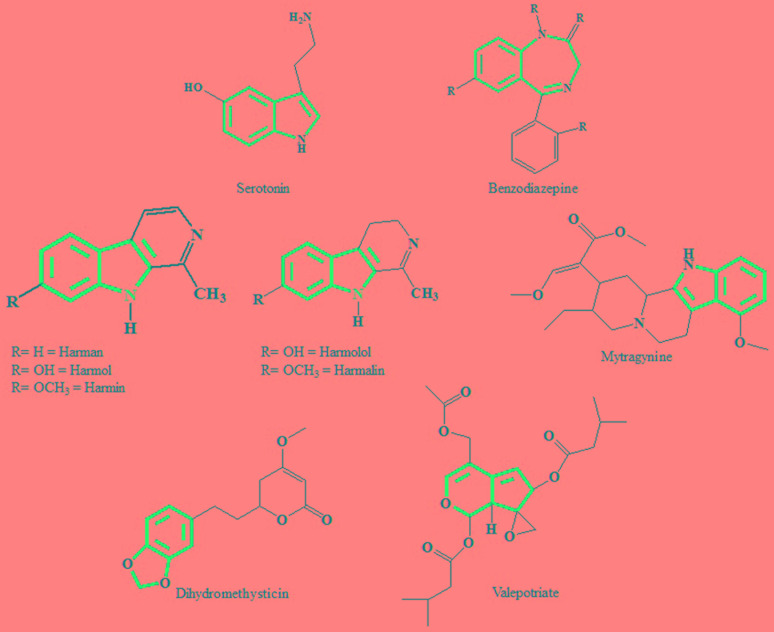
**Chemical structures of serotonin, benzodiazepine and isolated compounds from antidepressant plants.** Similarity in the skeleton pattern, which consists of six membered heterocyclic rings, attached with five membered rings.

At present the active constituents from plant extracts responsible for the antidepressant effect is still unclear. Thus we try to identify a few important chemical structures isolated from plant extracts exhibiting antidepressant activities and ascertain the skeleton pattern similarity that might contribute to antidepressant activity. Early attempts have been made to identify structural similarities between serotonin and indole alkaloids. The most distinct similarity consists of six membered heterocyclic rings fused to five membered rings. The difference is the presence of a nitrogen atom or distribution of π electrons (**Figure 1**). Through a structure activity relationship study Nichols (2012) reported that the variation in activity of different types of molecules suggest that the receptor is very sensitive to the nature of the tryptamine. Other chemical compounds that are successfully isolated from antidepressant plants are mentioned in this review paper because they are maybe specific agonists with particular substitution patterns that able to selectively activate a subset of effectors. This phenomenon is now known as functional selectivity.

**FIGURE 2:**
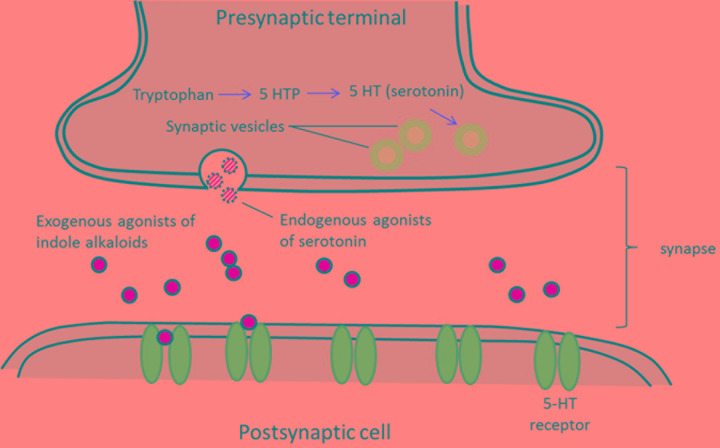
**Schematic of processes associated with neurotransmission.** Exogenous agonists of indole alkaloids from plants sharing structure similarities with serotonin that allows them to bind into serotonin receptors (5-HT receptors).

Serotonin, is widely used in brain function and cognition as endogenous receptor agonist (Fink and Göthert, 2007). Serotonin exerts its functions through seven families of receptors (5-HT1-5-HT7) which are members of the G-protein coupled receptor family. A number of compounds bearing the indole moiety have been described to own affinity toward different serotonin receptors (Kochanowska-Karamyan and Hamann, 2010). The structural similarity of indole alkaloids (exogenous agonists) to endogenous neurotransmitters like serotonin has led investigators to predict the potential neurological activity of these molecules. Schematic of processes associated with neurotransmission of exogenous agonists is described in **Figure 2**.

The indole ring is also known as bioisosteres and has similar chemical and physical as biological molecules. This similarity is used in the development of the prototype drug that aims to improve pharmacological activity and optimize the pharmacokinetic profile. In another study of pharmacological assessment of benzo[b]furans and thienopyrrole led to bioisosteric molecules that possess dimethyltryptamine-like activity. Early work with benzo[b]thiophenes and 3-indenalkylamines demonstrated that for compounds lacking ring substituents, the ability to act as agonists in the rat fundus was about the same as tryptamines. The results revealed the indole NH was not essential to activate the 5-HT2 receptor in the rat fundus (Nichols, 2012). A series of 2-aryl indole NK1 receptor antagonists and their derivatives are good ligands but have low oral bioavailability in rats. In order to increase solubility and absorption, the basic nitrogen was introduced, leading to the analog azaindole and related compounds exhibiting the same NK1 binding affinity with the series of 2-aryl indole NK1 receptor antagonists (Cooper et al., 2001). Molecular docking of 2 phenyl-indole derived ligands with serotonin 5-HT6 and melanocortin-4 receptors indicate that the privileged scaffold may accommodate depending on the nature conserved subpocket and non-conserved binding pocket. Interactions of non-conserved parts of the binding pocket are responsible for important differences in the molecular recognition by the corresponding target receptor (Bondensgaard et al., 2004).

According to de Sa et al. (2009) common indole alkaloids found in natural sources are tryptophan amino acids in human nutrition and the discovery of plant hormones that have therapeutic effects such as anti-inflammatory, a phosphodiesterase inhibitor, 5-HT receptor agonists and antagonists, cannabinoid receptor agonists and HMG-CoA reductase inhibitors. Indole scaffold has binding pockets and possesses common complementary binding domain to the target receptor, which belongs in a class of GPCRs (G-protein important membrane receptors coupled). Most drugs on the market contain the indole substructure. These include indomethacin, ergotamine, frovatriptan, ondansetron, and tadalafil.

## Conclusion

This review found a majority of the plant-based remedies indicated for the treatment of psychiatric ailments were crude or semipurified. The results for *in vivo* and *in vitro* varies and were not reproducible because in different biogeographical regions the secondary metabolite content of the plants correlates with availability of nutrients, climate and ecological conditions. In addition the bioactivity of plants might be contributed by a single compound or mixture of compounds. The authors suggest the effort to obtain active principles, phytochemicals identification, and metabolomics study should be conducted with inspections *in vitro* and *in vivo* for a better characterization of plant-based drugs. In most cases, the synthesis of indole alkaloids were inspired by the naturally occurring molecules and their similarity to serotonin.

The indole alkaloids from plant sources are quite complex compared to synthetic. The importance of synthetic indole alkaloids is already established as the structure is available in various ligand receptors, enzyme inhibitors and modulators bioreceptor. Some of the naturally occuring indole alkaloids cannot be synthesized by currently know methods. In addition, most of the information on the effectiveness of indole alkaloids was reported from synthetic indole alkaloids. As a result the potential of many naturally occuring indole alkaloids as new drug leads for various psychiatric disorders is still untapped. Historically, plant-based compounds have been the source of several of the most successful drug leads or drugs used in medicine. This is indicative that more could lay in store to be discovered.

In conclusion, several indole alkaloids have been employed as antidepressants or provide lead structures for its development. Based on our findings, plants contain a reservoir of indole alkaloids which are valuable starting points for the development of future antidepressants.

## Author Contributions

HH: Preparing manuscript. MY: Editing English language and suggest some important information in order to improve the manuscript. AR: Contribute for understanding the schematic of processes associated with neurotransmission.

## Conflict of Interest Statement

The authors declare that the research was conducted in the absence of any commercial or financial relationships that could be construed as a potential conflict of interest.
